# Supplementary material for: Diverse Begomoviruses Evolutionarily Hijack Plant Terpenoid-Based Defense to Promote Whitefly Performance
Source: Cells. 2022 Dec 30;12(1):149. doi: 10.3390/cells12010149 (PMC9818243; doi:10.3390/cells12010149)
Supplement: Supplementary file 1 [file cells-12-00149-s001.zip › cells-2076378-supplementary.pdf]

SUPPORTING INFORMATION

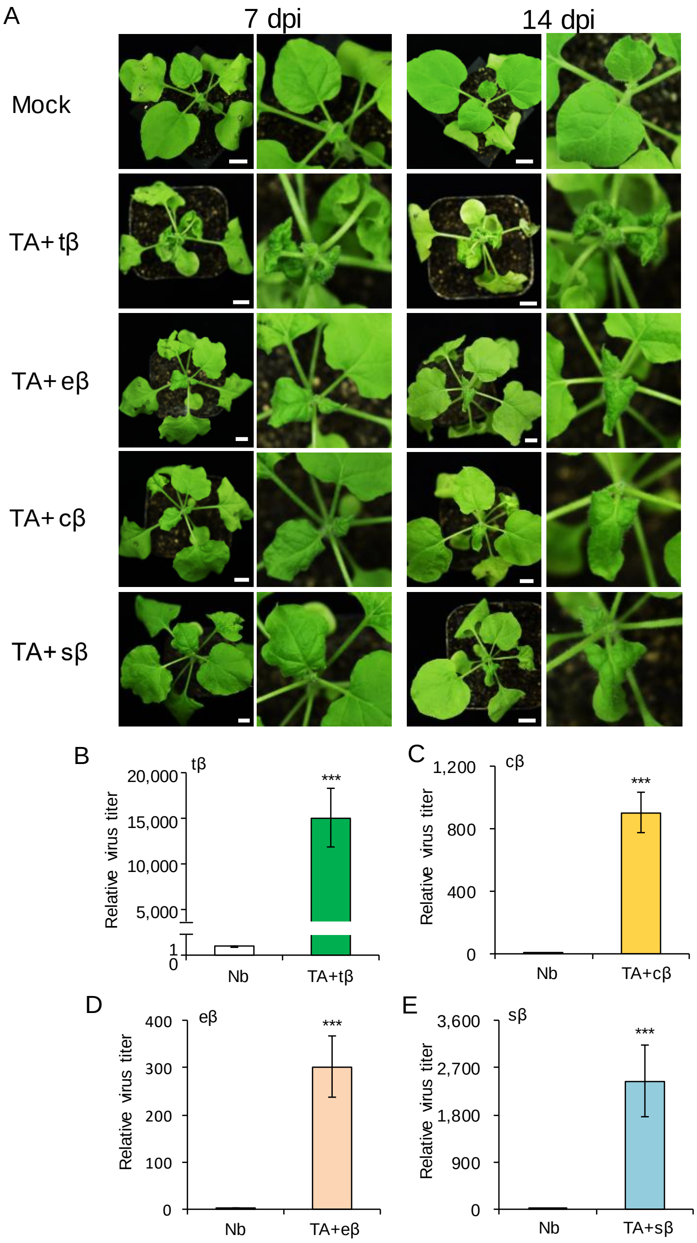

Figure S1. The betasatellites are required for begomovirus symptoms. (**A**) Symptoms of *N. benthamiana* (Nb) plants inoculated with TYLCCNV/TYLCCNB complex (TA+tβ), TYLCCNV/EPYVB complex (TA+eβ), TYLCCNV/CLCuMuB complex (TA+cβ) or TYLCCNV/SiYVB complex (TA+sβ) at 7dpi and 14 dpi. Nb inoculated with *Agrobacterium* was as mock (Nb, mock). Scale bars = 1 cm. (**B-E**) Viral DNA accumulation was quantified by RT-PCR in healthy Nb plants, and Nb plants infected by TA+tβ (**B**), TA+cβ (**C**), or TA+eβ (**D**), or TA+sβ (**E**). Bars represent means ± SD (n=4) (***, *P*< 0.001; Student's *t*-tests).

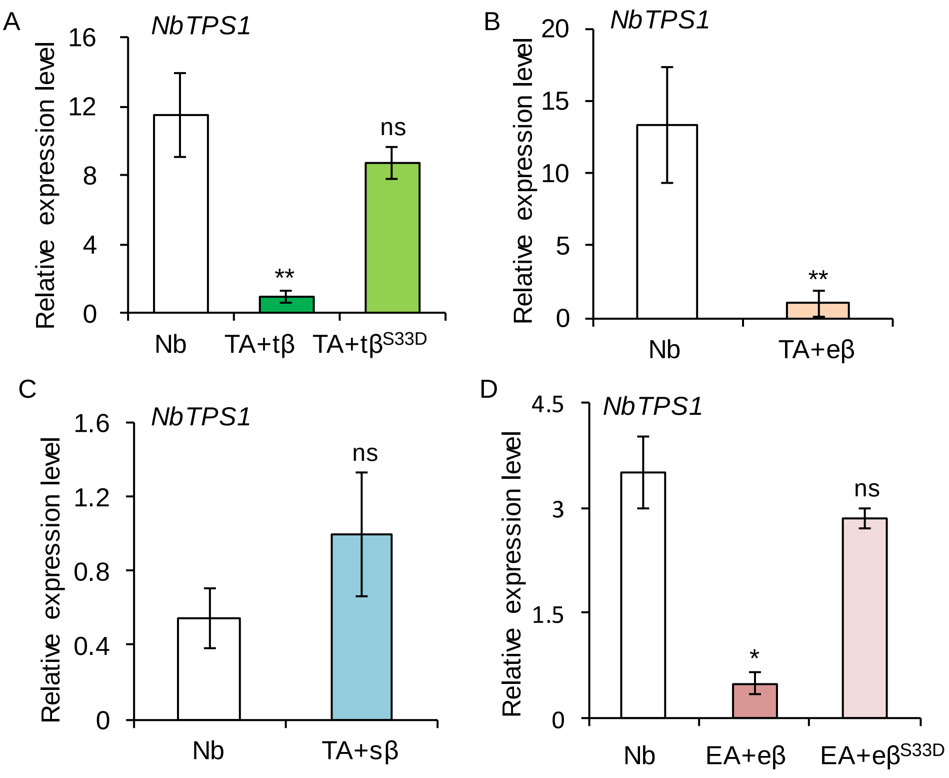

Figure S2. Begomovirus-betasatellite complex infection reduces *NbTPS1* expression. (**A**-**D**) Relative expression level of *NbTPS1* in healthy Nb plants, and Nb plants infected by TA+tβ and TA+tβ^{S33D} (**A**), TA+eβ (**B**), TA+sβ (**C**) and EA+eβ and EA+eβ^{S33D} (**D**). Bars represent means ± SD (n=4) (ns, no significant differences; *P < 0.05, **, P< 0.01; Student's t-tests).

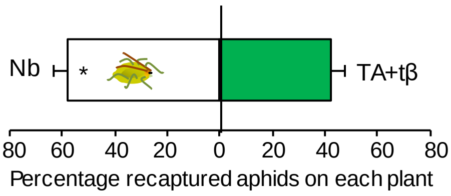

Figure S3. The TYLCCNV/TYLCCNB complex reduces plant attraction to non-vector aphids. The preference of non-vector aphids on uninfected *N. benthamiana* (Nb, mock) and plants infected by TA+tβ complex. Bars represent means + SD (n=6) (*P < 0.05; Wilcoxon matched pairs test).

**TYLCCNB**	T2, Y5, **S33**, T34, T78, Y110, T114, S116
ZiLCuB	T2, Y5, S6, T21, T22, S26, **S33**, T36, Y44, S65, Y74, S77, Y110
ToLCBaB	T2, Y5, S24, T32, **S33**, T34, S36, T77, T88, S104
TYLCVNB	T2, **S33**, T34, S51, S77, T78, T90
AlYVB	T4, **S33**, S40, S61, T78
**CLCuMuB**	S4, 6T, 8T, 14T, **S33**, T34, S36, S38, Y50, Y110, T117
MaYVYnB	S4, T14, **S33**, T34, S36, T38, T49, Y50, Y110, T117
CLCuBaB	T6, T7, T14, **S33**, T34, S36, S37, Y50, Y110, T117
**EpYVB**	Y5,T22, **S33**, Y40, Y48, T60, T88
**SiYVB**	T4, T14, S20, Y40, T43, T60, T67, T72, Y76, T81, T104, S109, T115

Figure S4. The potential phosphorylation sites of betasatellite-encoded βC1 proteins. Prediction of potential phosphorylation sites in some betasatellite-encoded βC1 proteins sequence using NetPhos 3.1.

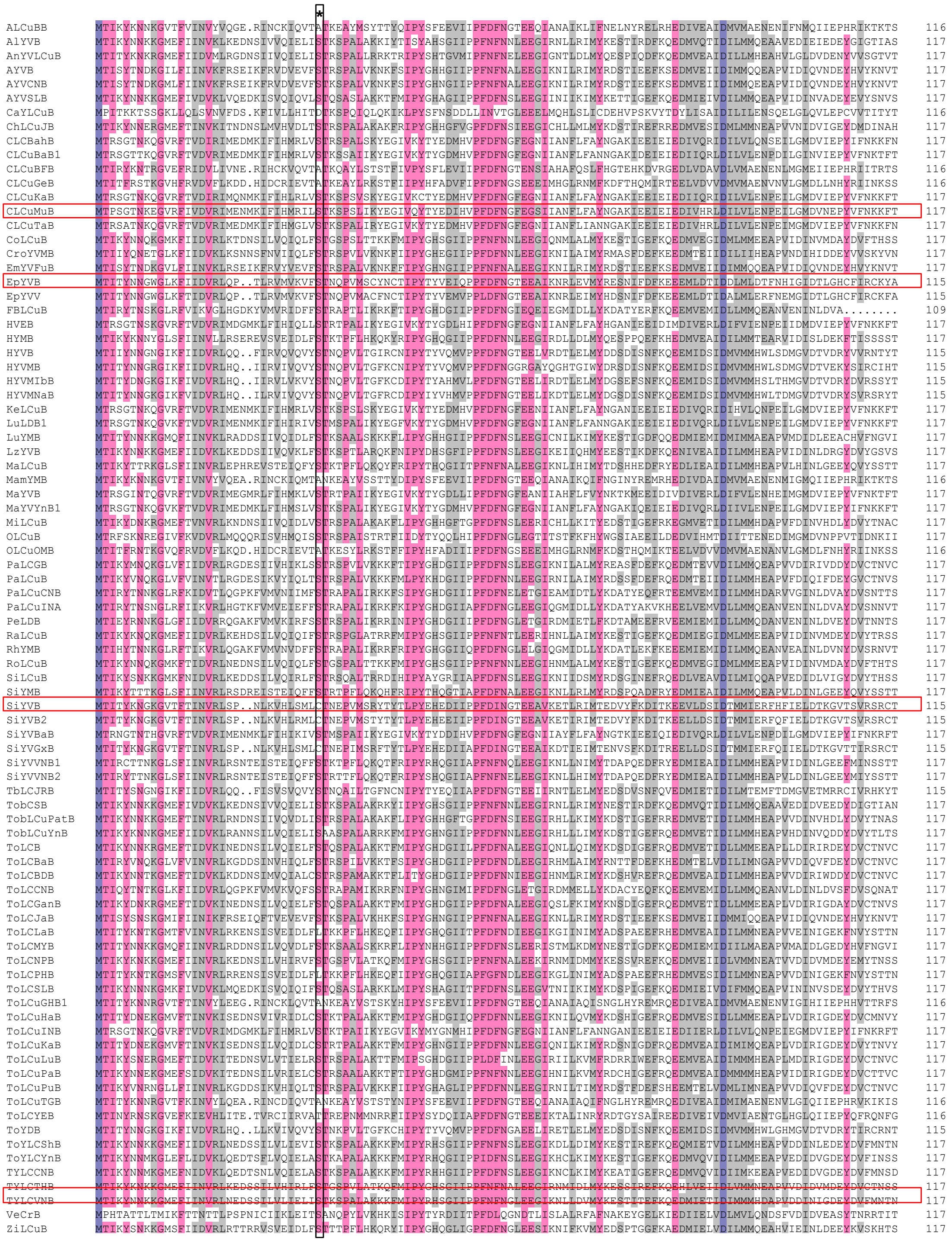

Figure S5. Serine-33 is the most conserved residue in βC1 proteins encoded by 119 betasatellite species. Multiple sequence alignments of βC1 proteins by DNAMAN. The asterisk indicates the most conserved phosphorylation site.

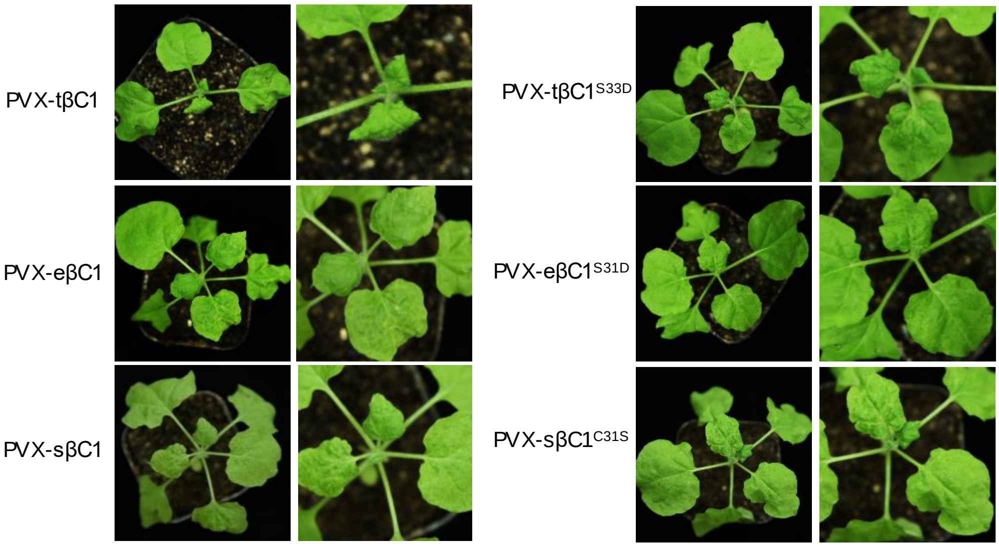

Figure S6. Symptoms of *N. benthamiana* inoculated with *Agrobacteria* carrying individual recombinant PVX vectors at 10 dpi. *N. benthamiana* leaves were inoculated with recombinant Potato virus X (PVX) vectors PVX-tβC1, PVX-tβC1^{S33D}, PVX-eβC1, PVX-eβC1^{S31D}, PVX-sβC1, PVX-sβC1^{C31S} via agroinfiltration.

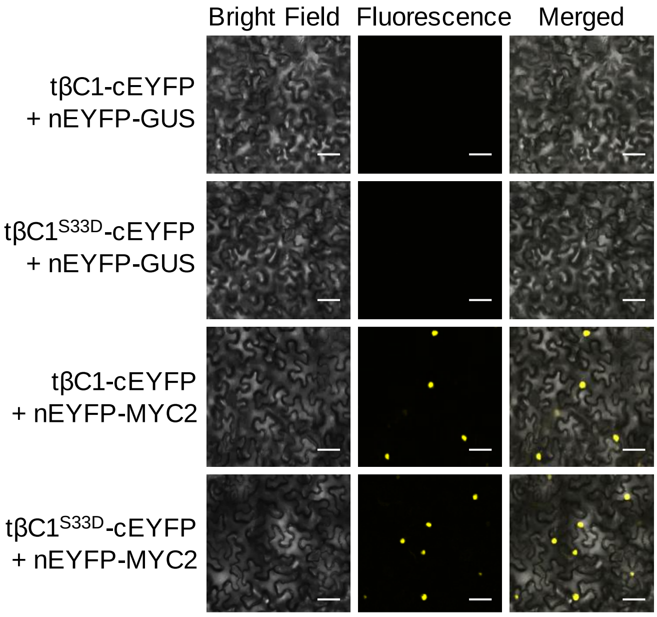

Figure S7. The phosphorylation mimic mutant βC1^{S33D} does not influence the interaction of βC1 with MYC2. BiFC analysis shows the interaction between wild-type tβC1 and MYC2 or the phosphorylation mimics mutant tβC1^{S33D} and MYC2. The nEYFP-GUS was used as a negative control. Scale bars = 50 μm.

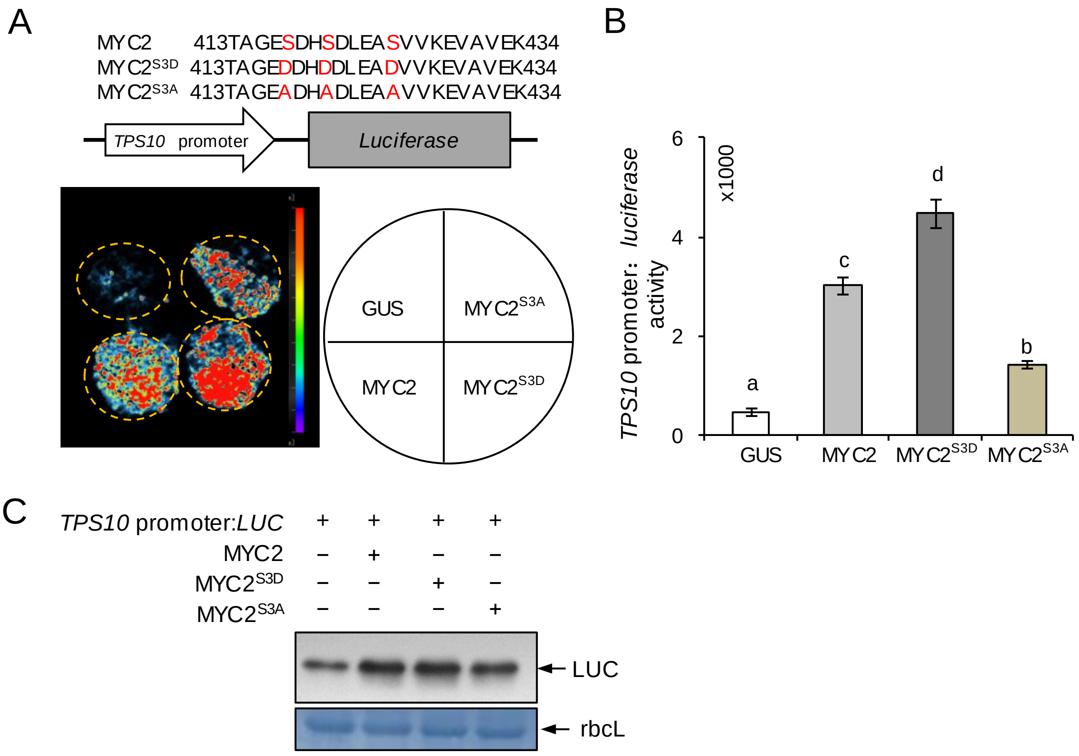

Figure S8. The phosphorylation levels of MYC2 influence its transcription activation. (**A**) Luciferase imaging of phosphorylation of MYC2 influencing its transcriptional activity on *TPS10* promoter in *N. benthamiana*. *N. benthamiana* leaves infiltrated *TPS10* promoter: *LUC* with *35S: MYC2*, low phosphorylation mutant *35S:* $MYC2^{S3A}$, phosphorylation mimic mutant *35S:* $MYC2^{S3D}$ or *35S: GUS* were subjected to luciferase complementation imaging assay. (**B**) Quantitative luminescence of *TPS10* promoter luciferase activity in *N. benthamiana*. *N. benthamiana* leaves infiltrated with indicated constructs were sliced into strips, and relative luminescence was determined by a microplate luminometer. Values are mean ± SEM (n = 8). Lowercase letters indicate significant differences among columns according to one-way ANOVA followed by Duncan's multiple range test ($P < 0.05$). (**C**) Western blot assay of LUC accumulation in agroinfiltrated leaf patches shown in figure B. Stained membrane bands of the large subunit of Rubisco (rbcL) were used as a loading control.

Name of species:		phosphorylation sites	location	Whitefly vector
Cardiospermum yellow leaf curl betasatellite	CaYLCuB	33D		
Sida yellow vein Madurai betasatellite	SiYVMaB	33L	Indian（southwest Asia)	
Ageratum leaf curl Buea betasatellite	ALCuBB	33A	Cameroon（west Africa)	
Ageratum leaf curl Cameroon betasatellite	ALCuCMB	33A	Cameroon（west Africa)	
Momordica yellow mosaic betasatellite	MamYMB	33A	Cameroon and Togo（west Africa)	
Tomato leaf curl Togo betasatellite	ToLCuTGB	33A	Togo（west Africa)	
Tomato leaf curl Ghana betasatellite1	ToLCuGHB1	33N	Ghana（west Africa)	
Tomato leaf curl Ghana betasatellite2	ToLCuGHB2	33A	Ghana（west Africa)	
Cotton leaf curl Burkina Faso betasatellite	CLCuBFB	33A	Burkina Faso（west Africa)	
Cotton leaf curl Gezira betasatellite	CLCuGeB	33A	Sudan（west Africa)	
Okra leaf curl Oman betasatellite	OLCuOMB	33A	Oman(southwest Asia)	
Tomato leaf curl Yemen betasatellite	ToLCYEB	33A	Yemen(southwest Asia)	
Tomato leaf curl Laguna betasatellite	ToLCLaB	33L	Philippines（southwest Asia)	
Chili leaf curl Sri Lanka betasatellite	ChLCuSLB	33A	Sri Lanka	
Tomato leaf curl Philippine betasatellite	ToLCPHB	33L	Philippine（southwest Asia)	
Vernonia yellow vein Fujian betasatellite	VYVFuB	33N	China	
Vernonia yellow vein betasatellite	VYVB	33R	Indian（southwest Asia)	
Siegesbeckia yellow vein betasatellite	SiYVB	33C	China	
Siegesbeckia yellow vein betasatellite 2	SiYVB2	33C	China	
Lindernia anagallis yellow vein betasatellite	LaYVB	33Y	Vietnam（southwest Asia)	
Siegesbeckia yellow vein Guangxi betasatellite	SiYVGxB	33C	China	

Figure S9. Variation analysis and location of betasatellite species which lost the conserved serine-33 site in βC1 proteins from the three subgroups. Location information is collected from NCBI genebank Description.

Table S1. Primers sequences used in this investigation.

Primers names	Primer Sequence (5'-3')	Purpose
PUC19-EPYVV-βC1-Flag-F	CGGGGGACGAGCTCGGTACC ATGACGATCACATACAAT	Cloning
PUC19-EPYVV-βC1-Flag-R	TGGTCTTTGTAGTCTTCGAA TACTGCGTATTTACATCG	Cloning
PUC19-SieYVV-βC1-Flag-F	CGGGGGACGAGCTCGGTACC ATGCACAAGATGACTATCAC	Cloning
PUC19-SieYVV-βC1-Flag-R	TGGTCTTTGTAGTCTTCGAA AAGTGTACATCTAGATCT	Cloning
PUC19-CLCuMuV-βC1-Flag-F	CGGGGGACGAGCTCGGTACC ATGACGAGGAGCAGAACA	Cloning
PUC19-CLCuMuV-βC1-Flag-R	TGGTCTTTGTAGTCTTCGAA AACGGTGAACTTCTTATT	Cloning
PUC19-TYLCCNV-βC1-Flag-F	CGGGGGACGAGCTCGGTACC ATGACTATCAAATACAAC	Cloning
PUC19-TYLCCNV-βC1-Flag-R	TGGTCTTTGTAGTCTTCGAA TACATCTGAATTTGTAAA	Cloning
TYLCCNV-βC1_Fw(Cal1)-PVX	CAAG ATCGAT ATGACTATCAAATACAAC	Cloning
TYLCCNV-βC1_Rv(Sal1)-PVX	CAAG GTCGAC TACATCTGAATTTGTAAA	Cloning
EPYVV-βC1_Fw(Cal1)-PVX	CAAG ATCGAT ATGACGATCACATACAAT	Cloning
EPYVV-βC1_Rv(Sal1)-PVX	CAAG GTCGAC TACTGCGTATTTACATCG	Cloning
SieYVV-βC1_Fw(Cal1)-PVX	CAAG ATCGAT ATGCACAAGATGACTATCAC	Cloning
SieYVV-βC1_Rv(Sal1)-PVX	CAAG GTCGAC AAGTGTACATCTAGATCT	Cloning
TYLCCNV-βC1_Fw(Cal1)-PVX	CAAG ATCGAT TGACTATCAAATACAACA	Cloning
TYLCCNV-βC1_Rv(Sal1)-PVX	CAAG GTCGAC TCATACATCTGAATTTGT	Cloning
CLCuMuV-βC1_Fw(Cal1)-PVX	CAAG ATCGAT ATGACGAGGAGCAGAACA	Cloning
CaLCuMV-βC1_Rv(Not1)-PVX	CAAG GCGGCCGC AACGGTGAACTTCTTATT	Cloning
SieYVB（C33S）-F	ATTTAAGCATGTTATCAACAAATGAACCAGTA	mutation
SieYVB（C33S）-R	TACTGGTTCATTTGTTGATAACATGCTTAAAT	mutation
EpYVB（S33D）-F	GTCAAGGTCTTCGACACCAATCAACCA	mutation
EpYVB（S33D）-R	TGGTTGATTGGTGTCGAAGACCTTGAC	mutation
QRT-TYLCCNV（βC1）-F	ATCCCACCATTCGACTTCAA	RT-qPCR
QRT-TYLCCNV（βC1）-R	TTCTACTGGGGCTTCTTCCA	RT-qPCR
QRT-CLCuMuV（βC1）-F	ATGACGAGGAGCAGAACA	RT-qPCR
QRT-CLCuMuV（βC1）-R	AACGGTGAACTTCTTATT	RT-qPCR
QRT-SieYVV（βC1）-F	AGCATGTTATGCACAAATG	RT-qPCR
QRT-SieYVV（βC1）-R	GCTGTCAAGTAGTTCTTCCTT	RT-qPCR

QRT-EpYVV（βC1）-F	CATACAATAACGGATGGGG	RT-qPCR
QRT-EpYVV（βC1）-R	CATCTATGGTGTCCAGCA	RT-qPCR
Nb -TPS1TPS1-F	TTAAAACGAACAAAAACAATACCCTCAT	RT-qPCR
Nb -TPS1TPS1-R	CTCTTGAGCATACATTTGTGCAACC	RT-qPCR
Nb -EF1αEF1α-F	TGGTGTCCTCAAGCCTGGTATGGTTG	RT-qPCR
Nb -EF1αEF1α-R	ACGCTTGAGATCCTTAACCGCAACATTCTT	RT-qPCR

Table S2. Betasatellite accession numbers from ICTV Report

**Species name**	**Virus acronym**	**Acc. No.**
Honeysuckle yellow vein mosaic betasatellite	HYVMB-[JR-Hy-04]	AB182263
Tobacco leaf curl Japan betasatellite	TbLCJRB-[JR-Miy-05]	AB236324
Honeysuckle yellow vein mosaic Ibaraki betasatellite	HYVMIbB	AB287442
Honeysuckle yellow vein mosaic Nara betasatellite	HYVMNaB	AB287443
Tomato yellow dwarf betasatellite	ToYDB	AB294512
Eupatorium yellow vein mosaic betasatellite	EpYVV-[JR-Suya-03]	AB300464
Tomato leaf curl Laguna betasatellite	ToLCLaB-[PH-Lag2-06]	AB307732
Tomato leaf curl Philippine betasatellite	ToLCPHB-[PH-Lag1-06]	AB308071
Ageratum yellow vein betasatellite	AYVB-[SG-95]	AJ252072
Cotton leaf curl Multan betasatellite	CLCuMuB-[PK-Mul-U89-97]	AJ298903
Bhendi yellow vein mosaic betasatellite	BYVB-[IN-Mut-00]	AJ308425
Ageratum yellow leaf curl betasatellite	AYLCB-[PK-Fai4-00]	AJ316026
Chili leaf curl betasatellite	ChLCuB-[PK-MC-97]	AJ316032
Tomato leaf curl betasatellite	ToLCB-[PK-RYK-97]	AJ316036
Honeysuckle yellow vein betasatellite	HYVB-[UK-Nor1-99]	AJ316040
Tomato yellow leaf curl China betasatellite	TYLCCNB-[CN-Yn45-01]	AJ420313
Tobacco curly shoot betasatellite	TobCSB-[CN-Yn35-01]	AJ421484
Eupatorium yellow vein betasatellite	EpYVB-[JR-MNS2-00]	AJ438938
Tomato leaf curl Bangladesh betasatellite	ToLCBDB-[BD-Gaz-01]	AJ542489
Tomato leaf curl Laos betasatellite	ToLCLAB-[LA-Sav-01]	AJ542491
Tomato leaf curl Nepal betasatellite	ToLCNPB-[NP-Jhapa]	AJ542492
Tomato leaf curl Sri Lanka betasatellite	ToLCSLB-[SL]	AJ542493
Ageratum yellow vein Sri Lanka betasatellite	AYVSLB-[SL-Ag-03]	AJ542498
Zinnia leaf curl betasatellite	ZiLCuB	AJ542499
Tomato yellow leaf curl Thailand betasatellite	TYLCTHB-[CN-Yn72-02]	AJ566746
Tomato leaf curl China betasatellite	ToLCCNB-[CN-Gx14-02]	AJ704609
Malvastrum yellow vein Yunnan betasatellite 1	MaYVYnB1	AJ786712
Sida yellow mosaic betasatellite	SiYMB	AJ810093
Ludwigia yellow vein betasatellite	LuYMB	AJ965541

Tomato leaf curl Joydebpur betasatellite	ToLCJoB-[BD-Gaz-05]	AJ966244
Sida yellow vein Madurai betasatellite	SiYVMaB	AJ967003
Ageratum yellow vein China betasatellite	AYVCNB	AJ971257
Malvastrum yellow vein betasatellite	MaYVB	AJ971459
Sida leaf curl betasatellite	SiLCuB	AM050732
Malvastrum leaf curl betasatellite	MaLCuB-[CN-Gx87-04]	AM072289
Siegesbeckia yellow vein Guangxi betasatellite	SiYVGxB	AM238695
Tobacco leaf curl betasatellite	TobLCuB-[PK-Lah-04]	AM260465
Croton yellow vein mosaic betasatellite	CroYVMB-[PK-Pun-06]	AM410551
Cardiospermum yellow leaf curl betasatellite	CaYLCuB-[SL-04]	AM933578
Papaya leaf curl betasatellite	PaLCuB-[IN-ND-03]	AY244706
Tomato leaf curl Bangalore betasatellite	ToLCBaB-[IN-Ban-03]	AY428768
Cotton leaf curl Bangalore betasatellite 1	CLCuBaB1	AY705381
Ludwigia leaf distortion betasatellite 1	LuLDB1	AY728262
Tomato leaf curl Pune betasatellite	ToLCuPuB	AY754815
Sida yellow vein Vietnam betasatellite 1	SiYVVNB1	DQ641712
Erectites yellow mosaic betasatellite	ErYMB	DQ641713
Tomato yellow leaf curl Vietnam betasatellite	TYLCVNB-[VN-Han-05]	DQ641714
Lindernia anagallis yellow vein betasatellite	LaYVB	DQ641715
Alternanthera yellow vein betasatellite	AlYVB-[VN-Hue-05]	DQ641716
Cotton leaf curl Gezira betasatellite	CLCuGeB-[SD-Dat-06]	DQ644564
Radish leaf curl betasatellite	RaLCuB	EF175734
Ludwigia leaf distortion betasatellite 2	LuLDB2	EF614160
Cotton leaf curl Bahraich betasatellite	CLCBahB	EF620566
Sida yellow vein Barrackpore betasatellite	SiYVBaB	EU188921
Tomato leaf curl Bangalore betasatellite 2	ToLCuBaB2	EU280314
Tomato leaf curl Patna betasatellite	ToLCPaB-[IN-Pat-07]	EU862324
Emilia yellow vein betasatellite	EmYVB	FJ869906
Ageratum leaf curl Cameroon betasatellite	ALCuCMB-[CM-Man-AMBF-06]	FM164737
Vernonia yellow vein betasatellite	VYVB-[IN-Mad-09]	FN435836
Kenaf leaf curl betasatellite	KeLCuB	FN678779
Malvastrum yellow vein Yunnan betasatellite 2	MaYVYnB1	FN806780
Ageratum leaf curl Buea betasatellite	ALCuBB-[CM-LIO1-SatB33-09]	FR717140
Leucas zeylanica yellow vein betasatellite	LzYVB	GQ421324
Rose leaf curl betasatellite	RoLCuB-[PK-Fai-06]	GQ478344
Tomato leaf curl Ranchi betasatellite	ToLCuRaB	GQ994096
Okra leaf curl betasatellite	OLCuB	GU111963
Chili leaf curl Jaunpur betasatellite	ChLCuJB-[IN-Jau-07]	HM007103
Papaya leaf curl India betasatellite	PaLCuINA-[India-Panipat-08]	HM143906
Tomato leaf curl Panipat betasatellite	ToLCuPaB	HM143907
Tobacco leaf curl Patna betasatellite	TobLCuPatB-[IN-Pusa-09]	HQ180394
Tomato leaf curl Togo betasatellite	ToLCuTGB	HQ586965
Siegesbeckia yellow vein betasatellite 2	SiYVB2	JF682839

Vernonia yellow vein Fujian betasatellite	VYVFuB-[CN-09]	JF733779
Tomato leaf curl Yemen betasatellite	ToLCYEB-[YE-tob56-89]	JF919717
Chili leaf curl Sri Lanka betasatellite	ChLCuSLB-[SL-Mih-09]	JN638445
Tomato leaf curl India betasatellite	ToLCuINB	JQ012916
French bean leaf curl betasatellite	FBLCuB-[IN-Kan-11]	JQ866298
Tobacco leaf chlorosis betasatellite	TobLCB	JX025223
Tomato leaf curl Hajipur betasatellite	ToLCuHaB	JX262390
Mungbean yellow mosaic betasatellite	MYMB-[IN-Cowpea-12]	JX443646
Tomato leaf curl Java virus betasatellite	ToLCJaB-[NP-R7-Papaya-2010]	KC282642
Tobacco leaf curl Yunnan betasatellite	TobLCuYnB	KC699042
Tomato leaf curl Gandhinagar betasatellite	ToLCGanB-[IN-pToGNbH14-12]	KC952006
Andrographis yellow vein leaf curl betasatellite	AnYVLCuB-[IN-Luc-10]	KC967282
Okra leaf curl Oman betasatellite	OLCuOMB-[OM-Barka-12]	KF267444
Siegesbeckia yellow vein betasatellite	SiYVB-[CN-FZ02-12]	KF499590
Tomato yellow leaf curl Yunnan betasatellite	ToYLCYnB-[CN-tob-]	KF640694
Hedyotis yellow mosaic betasatellite	HYMB-[VN-BinhDinh-13]	KF641186
Cotton leaf curl Bangalore betasatellite 2	CLCuBaB2	KF964654
Sida yellow vein Vietnam betasatellite 2	SiYVVNB2	KF990602
Papaya leaf curl China betasatellite	PaLCuCNB-[CN-Hainan-14]	KJ642219
Tomato leaf curl Malaysia betasatellite	ToLCMYB-[MY-13]	KM051528
Malvastrum yellow vein Cambodia betasatellite	MaYVKHB	KP188832
Tomato yellow leaf curl Shandong betasatellite	ToYLCShB-[CN-SDSG-14]	KP322555
Rhynchosia yellow mosaic betasatellite	RhYMB-[IN-Pha-14]	KP752092
Papaya leaf curl Gandhinagar betasatellite	PaLCGB	KT253638
Tomato leaf curl Ghana betasatellite 1	ToLCuGHB1	KT382328
Tomato leaf curl Ghana betasatellite 2	ToLCuGHB2	KT382329
Ludwigia leaf distortion betasatellite 3	LuLDB3	KT390358
Momordica yellow mosaic betasatellite	MamYMB-[BJ-57-14-14]	KT454829
Tomato leaf curl betasatellite 2	ToLCuB2	KU500806
Vernonia crinkle betasatellite	VeCrB	KX831134
Pea leaf distortion betasatellite	PeLDB	KY001644
Cotton leaf curl Bangalore betasatellite 3	CLCuBaB3	LC316186
Mirabilis leaf curl betasatellite	MiLCuB-[IN-Him-13]	LK054803
Cotton leaf curl Kashmir betasatellite	CLCuKaB	LN610993
Tomato leaf curl Pakistan betasatellite	ToLCuPKB	LN811050
Tobacco leaf curl Sheikhupura betasatellite	TobLCuShB	LT795119
Cotton leaf curl Tandojam betasatellite	CLCuTaB	LT827054
Hibiscus vein enation betasatellite	HVEB	MF140456
Codiaeum leaf curl betasatellite	CoLCuB	MF278784
Tomato leaf curl Lucknow betasatellite	ToLCuLuB	MG478451
Tomato leaf curl Joydebpur betasatellite 2	ToLCuJoB2	MG571522
Tomato leaf curl Karnataka betasatellite	ToLCuKaB	MG758146
Cotton leaf curl Bangalore betasatellite 4	CLCuBaB4	MG758149

Emilia yellow vein Fujian betasatellite	EmYVFuB	MH035671
Tomato leaf curl Bundi betasatellite	ToLCuBuB	MH577020
Cotton leaf curl Burkina Faso betasatellite	CLCuBFB	MK032307
Papaya leaf curl India betasatellite 2	PaLCINB2	MN529627
